# Enhanced Recovery after Surgery (ERAS) for Hip and Knee Replacement—Why and How It Should Be Implemented Following the COVID-19 Pandemic

**DOI:** 10.3390/medicina57010081

**Published:** 2021-01-19

**Authors:** Thomas W. Wainwright

**Affiliations:** 1 Orthopaedic Research Institute, Bournemouth University, 6th Floor, Executive Business Centre, 89, Holdenhurst Road, Bournemouth BH8 8EB, UK; twainwright@bournemouth.ac.uk; Tel.: +44-01202-961656; 2 Physiotherapy Department, University Hospitals Dorset NHS Foundation Trust, Bournemouth BH7 7DW, UK

**Keywords:** enhanced recovery after surgery, hip replacement, knee replacement, COVID-19, outpatient surgery

## Abstract

The COVID-19 pandemic has led to a reduction in hip and knee replacement surgery across healthcare systems. When regular operating returns, there will be a large volume of patients and an emphasis on a short hospital stay. Patients will be keen to return home, and capacity will need to maximised. Strategies to reduce the associated risks of surgery and to accelerate recovery will be needed, and so Enhanced Recovery after Surgery (ERAS) should be promoted as the model of care. ERAS protocols are proven to reduce hospital stay safely; however, ERAS pathways may require adaption to ensure both patient and staff safety. The risk of exposure to possible sources of COVID-19 should be limited, and so hospital visits should be minimised. The use of technology such as smartphone apps to provide pre-operative education, wearable activity trackers to assist with rehabilitation, and the use of telemedicine to complete outpatient appointments may be utilised. Also, units should be reminded that ERAS protocols are multi-modal, and every component is vital to minimise the surgical stress response. The focus should be on providing better and not just faster care. Units should learn from the past in order to expedite the implementation of or adaption of existing ERAS protocols. Strong leadership will be required, along with a supportive organisational culture, an inter-professional approach, and a recognised QI method should be used to contextualize improvement efforts.

## 1. Introduction

The COVID-19 pandemic has and will continue to have a significant impact on healthcare systems across the world. Whilst the current focus is to deal with the acute effects of the pandemic, there will be subsequent pressures felt within our healthcare systems. These include the long-term rehabilitation of COVID-19 patients; the management of the sequelae of interrupted care for patients with long-term chronic conditions; and safely resuming elective surgery for an increased number of patients [1].

For the surgical specialities, a phased return to elective procedures will be seen. Within orthopaedic surgery, this will mean considerable challenges for patients with hip and knee osteoarthritis who require replacement surgery. Due to the non-life-threatening nature of osteoarthritis, the age of patients awaiting surgery (commonly over 65 years old), and the in-hospital stay usually required following the procedure, it will mean that hip and knee replacement surgeries will be some of the last surgical procedures recommended to return [2]. Therefore, when patients present for surgery, they are likely to have increased disability levels, be more de-conditioned (following reduced activity levels due to social isolation and also increased pain due to the prolonged wait for surgery), and there will be a large volume of patients to treat [3]. Strategies will be needed to reduce the associated risks of surgery and to accelerate recovery, at the same time as optimising capacity. Enhanced Recovery after Surgery (ERAS) protocols can deliver these outcomes [4] and should, along with outpatient surgery (where suitable and safe), be promoted as the model of care [5].

## 2. Enhanced Recovery after Surgery (ERAS)

ERAS programs (or Rapid Recovery or Fast-track programs) have developed over the past 20 years. They have been demonstrated to reduce length of hospital stay (LOS), morbidity, and convalescence time, without an increase to readmission rates or complications [6]. ERAS protocols can be considered a Quality Improvement (QI) intervention, and are an inter-professional and multi-modal approach to care. ERAS protocols seek to optimise patient care before, during, and after surgery in order to minimise the surgical stress response. They are multi-modal and combine techniques such as pre-operative education, minimally invasive surgery, regional anaesthetic techniques, multimodal opioid-sparing pain management, and early mobilisation. ERAS protocols have been detailed and summarised for total hip replacement and total knee replacement [4]. ERAS protocols are being promoted as key strategies to be adopted by orthopaedic clinical microsystems when hip and knee replacement is restarted, and it is anticipated that there may be an increase in early discharge and day-case (or outpatient) surgery [3].

## 3. Nursing and Allied Health Professional (AHP) Adaptations to ERAS Protocols Following COVID-19

ERAS pathways will require some adaption as surgery returns following the COVID-19 pandemic in order to ensure both patient and staff safety. Whilst the actual surgery and anaesthetic will remain relatively unchanged (there may well be some changes to facilitate more outpatient surgery e.g., timing in the day of surgery, and shorter acting anaesthetics), nursing and AHP interventions pre- and post-operatively are likely to be significantly adapted. Clinical microsystems will be required to adhere to new evidence-based practices to risk-stratify patients before surgery, screen for COVID-19, and utilise strategies to minimise possible exposure whilst in hospital (e.g., Personal Protective Equipment (PPE) requirements for both patients and healthcare workers). Such changes to practice will be general principles applicable to all surgery. They should be made in accordance with the relevant local policy regarding the surgical management of patients post COVID-19 pandemic.

With specific relevance to ERAS protocols, in order to minimise risk, patients will need to be discharged from hospital back home as soon as it is safely possible to do so. Patients are likely to be strongly motivated to get home and distance themselves from exposure to COVID-19, but this should be done safely. Patients will still need to achieve the required medical, nursing, and therapy milestones in order to be discharged without a risk of increased complications and readmission. Caution is required because recent data has indicated that there may be an increased risk of complications for patients discharged on the day of surgery compared to those who stay in the hospital for 1–2 days [7]. Therefore, careful pre-operative discharge planning by nurses and therapists will be essential, with provisions made for self-isolating or shielding following surgery (if required by local policy). In addition, at the pre-operative stage, patients with substantial surgical risk factors should be optimised by ERAS protocols to reduce the chance of post-operative complications [4]. For those patients with non-modifiable risk factors in relation to surgery and also COVID-19, an informed decision making process should be undertaken in partnership with the patient, so that conservative treatment options and delayed surgery are considered as alternatives.

The potential role of utilising technology in ERAS pathways has previously been highlighted [8], and its use may offer advantages for nurses and AHPs at multiple stages of the pathway, post COVID-19. For example, the provision of pre-operative information and education is often delivered to patients before hip and knee replacement via a pre-operative class or “joint school”. Alternative options can be made available via online resources or smartphone apps. If hospitals do not already have a smartphone app, generic apps are available and can be utilised [9]. Also, wearable devices and activity trackers may be utilised post-operatively for patients to manage their rehabilitation independently [10], and post-discharge follow up check-ups can be conducted via telephone or video follow up [11]. Whilst conducting remote follow up, patients and carer’s must be informed of how to contact appropriate services if they are concerned about the development of complications in between appointments, and these communication channels should be integrated with community and primary care teams.

The need and importance of a consistent and seven-day provision of therapy has been previously highlighted within ERAS pathways [12] and must continue, or be established. Daily inter-professional ward rounds with senior nursing and AHP presence will be required, so that any barriers to discharge can be assessed and acted on quickly so that delays to discharge are minimised.

## 4. Ensuring the Successful Implementation and Adaptation of ERAS Pathways

The importance of highlighting the role of implementing ERAS when surgery returns following COVID-19 is two-fold. First, despite the evidence-base and published clinical guidelines [4], the widespread global implementation of ERAS is not complete. In many hospitals, ERAS is not yet the standard of care. For example, LOS is still around four days after hip and knee replacement in countries such as England and Spain [13,14], compared with 0–2 days in extensive epidemiological studies from Denmark and USA [7,15]. The restarting of services, therefore represents an opportunity to “reset” pathways at a time when limited capacity and increased demand may help to drive positive changes. Second, for those sites with ERAS already implemented, there will be a push to progress towards day-case or outpatient surgery in order to further maximise resources, and the need to adapt existing protocols to incorporate digital solutions (as described previously) will bring an additional quality improvement challenge.

In both cases, ERAS teams can learn from the past in order to expedite the implementation of or adaption of existing ERAS protocols, so that insights from previous implementation are taken advantage of. There may also be new opportunities following COVID-19 to make improvements. For example, the organisational need to maximise capacity and resources may provide economic levers to change as well as challenges depending on the context. Also, the response to the COVID-19 pandemic has required inter-professional and cross-departmental working in many hospitals, and these strengthened relationships have allowed our healthcare systems to change at speed. There may be an opportunity for surgical units to build on this inter-professional collaboration, an essential factor, given that the role of good teamwork and integrated working is a known element of high performing hip and knee ERAS units [16].

It should also be remembered that ERAS is a QI intervention, and so if the QI literature is looked at more broadly further insight and confirmation of critical contextual success factors can be found. Eight key contextual factors linked to the success of QI efforts have recently been proposed following a systematic realist review [17]. These factors are:

1. Active, supportive, and engaged leadership
2. Multi-disciplinary collaboration
3. A supportive organisational culture
4. Staff with the right individual skills and capabilities
5. Organisational capacity and capability for QI
6. An infrastructure to collect and analyse outcome data
7. A shared readiness and belief in change
8. A change agent to drive and lead the change

These factors resonate with the ERAS implementation literature [18,19] and present an “aide memoir” for health professionals tasked with implementing change to or the introduction of an ERAS pathway. For those leading the change, the concepts outlined by [17] should be used to enhance the planning of any QI effort.

Given this knowledge and context, orthopaedic teams seeking to implement or adapt their ERAS pathway should therefore ensure that their clinical and managerial leaders recognise that they will need to actively lead and take responsibility for the change at all levels. They should recruit a change agent, drive the improvement of organisational characteristics if required (such as QI knowledge, skills and capability) and seek to create a supportive organisational culture, to ensure that staff recognise the benefits of changes for patients and are motivated to change. Support from administrators will also be needed to help with the data and technical infrastructure to support outcome monitoring and digital solutions.

With these components in place, the use of a recognised QI method to ensure the correct changes are made to the care process for the local context is also recommended. This is because even though ERAS pathways have been proven to improve clinical outcomes, their delivery is context-dependent, and as they are developed, changes need to be holistically informed. Improving a clinical outcome is achieved by combining clinical decisions informed by evidence-based medicine (such as an ERAS protocol) with the needed process or system changes, that allow the right things to be delivered in the right way [20]. Understanding this concept is crucial, and when combined with effective leadership, a supportive organisational culture, and an infrastructure to collect data, units will be able to understand how to improve their pathways.

## 5. Conclusions

Following the COVID-19 pandemic, there will be a large volume of hip and knee replacement patients and an emphasis on a short hospital stay. Therefore, ERAS should be promoted as the model of care. However, ERAS pathways may require adaption by orthopaedic teams at the pre and post-operative stages to ensure patient and staff safety. The use of technologies such as smartphone apps, wearable activity trackers, and telemedicine may be utilised so that the focus remains on providing better and not just faster care. When adapting pathways, inter-disciplinary teams should learn from the past, and recognised that strong leadership, a supportive organisational culture, and the use of a recognised QI method will be required to contextualise and ensure successful improvement efforts.
